# Alpha fetoprotein plays a critical role in promoting metastasis of hepatocellular carcinoma cells

**DOI:** 10.1111/jcmm.12745

**Published:** 2016-01-12

**Authors:** Yan Lu, Mingyue Zhu, Wei Li, Bo Lin, Xu Dong, Yi Chen, Xieju Xie, Junli Guo, Mengsen Li

**Affiliations:** ^1^ Hainan Provincial Key Laboratory of Carcinogenesis and InterventionHainan Medical CollegeHaikouHainan ProvinceChina; ^2^ Key Laboratory of Molecular BiologyHainan Medical CollegeHaikouHainan ProvinceChina; ^3^ Department of PathophysiologyHainan Medical CollegeHaikouHainan ProvinceChina; ^4^ Institution of TumorHainan Medical CollegeHaikouHainan ProvinceChina

**Keywords:** alpha fetoprotein, liver cancer cells metastasis, therapeutic target

## Abstract

A high level of serum alpha fetoprotein (AFP) is positively associated with human hepatocellular carcinoma (HCC) carcinogenesis and metastasis; however, the function of AFP in HCC metastasis is unknown. This study has explored the effects of AFP on regulating metastatic and invasive capacity of human HCC cells. Forty‐seven clinical patients' liver samples were collected and diagnosed; HCC cells line, Bel 7402 cells (AFP‐producing) and liver cancer cell line cells (non‐AFP‐producing) were selected to analyse the role of AFP in the metastasis of HCC cells. The results indicated that high serum concentration of AFP was positively correlated with HCC intrahepatic, lymph nodes and lung metastasis. Repressed expression of AFP significantly inhibited the capability of migration and invasion of Bel 7402 cells, expression of keratin 19 (K19), epithelial cell adhesion molecule (EpCAM), matrix metalloproteinase 2/9 (MMP2/9) and CXC chemokine receptor 4 (CXCR4) were also down‐regulated in Bel 7402 cells; migration and invasion, expression of K19, EpCAM, MMP2/9 and CXCR4 were significantly enhanced when HLE cells were transfected with AFP‐expressed vector. The results demonstrated that AFP plays a critical role in promoting metastasis of HCC; AFP promoted HCC cell invasion and metastasis *via* up‐regulating expression of metastasis‐related proteins. Thus, AFP may be used as a novel therapeutic target for treating HCC patients.

## Introduction

Hepatocellular carcinoma (HCC) is the fifth of common cancers and the third of leading causes of cancer deaths worldwide 1. As a common malignant neoplasm and a cause of cancer‐related death in Asia and Africa, HCC is associated with a high rate of mortality due to lack of effective treatments against HCC invasion and metastasis 2, 3. Metastasis has become the major obstacle to survival and quality of life in HCC patients 4. Recently, we have found that alpha fetoprotein (AFP), a specific biomarker of HCC, harbours a function to activate phosphatidylinositol 3‐kinase (PI3K)/protein kinase B (AKT) signal pathway to stimulate expression of metastasis‐related factor, such as CXC motif chemokine receptor 4(CXCR4) 5. Moreover, high serum level of AFP might be used as a predictor for HCC metastasis and poor clinical outcomes 6. These facts implicated that AFP may play a role in the metastasis of HCC.

In the early stage of hepatocarcinogenesis, *AFP* gene is reactivated in liver cells; cytoplasmic AFP promoted malignant liver cells proliferation through stimulating expression of Src, c‐myc 7. Extracellular AFP also accelerates growth of HCC cells that is mediated by AFP receptor 8. Liver cancer cells possess malignant biology behaviours, including metastasis. The metastasis of HCC involves in elevating expression of metastasis‐related molecules, including keratin 19 (K19) 9, epithelial cell adhesion molecules (EpCAM) 10, matrix metalloproteinase 2/9 (MMP2/9) 11 and CXCR4 12 in hepatoma cells. Expression of these genes is regulated by PI3K/AKT signal pathway 13, 14, 15, 16. Although investigations have discovered that AFP activation of PI3K/AKT signal pathway through inhibiting activity of phosphatase and tensin homolog deleted on chromosome ten (PTEN) 17, and high expression of AFP positively associated with metastasis of HCC cells, biological effect of AFP on promoting metastasis of HCC cells is still unknown. In this study, we investigated the effects of AFP on metastasis of HCC cells. The results indicated that AFP directly to promote metastasis of HCC cells *via* stimulating expression of metastasis‐related genes, K19, EpCAM, MMP2/9 and CXCR4. Thus, AFP could be applied as a novel therapeutic target for confronting HCC invasion and metastasis.

## Material and methods

### Patients and specimens

The archived clinical specimens were originally collected during hepatectomy of 47 patients, including six cases of liver trauma patients (normal liver specimens) and 41 cases of HCC specimens (diagnosis confirmed 16 cases: non‐metastasis and 25 cases: metastasis) at Hainan Provincial People's Hospital (Haikou, Hainan, China) and the Affiliated Hospital of the Hainan Medical College (Haikou, Hainan, China) between January 2010 and November 2013. Of the 47 patients, 32 men and 15 women with an average age of 50.8 (range 31–77) years. All enrolled patients were treated with radical surgery and received no other treatments. Circulating AFP serum level was measured by ELISA. Clinical data were obtained by a retrospective chart review. Follow‐up was available for all patients. A section of liver tissue about 2.0 × 2.0 × 2.0 cm was obtained from each patient immediately after the surgery. About 1.0 × 1.0 × 1.0 cm tissue samples were fixed in 10% formalin, embedded in paraffin and routinely stained with hematoxylin and eosin. The 1.0 × 1.0 × 1.0 cm tissue specimens were stored in liquid nitrogen. All of specimens were assessed blindly and independently by two pathologists. In case of interobserver disagreement, final decisions were achieved by general consensus. All selected patients were diagnosed by histopathological evaluation and metastasis of HCC patients was estimated by computerized tomography (CT). The study protocol was approved by the Ethical Committee of Hainan Provincial People's Hospital and the Science Investigation Ethical Committee of Hainan Medical College. Written informed consent was obtained from all participants.

### Immunohistochemical analysis

The expression and cellular distribution of AFP and CXCR4 proteins in HCC specimens were assessed by immunohistochemical analysis. Five‐millimetre‐thick paraffin sections were deparaffinized and rehydrated according to standard protocols, and heat‐induced antigen retrieval was performed in sodium citrate buffer (10 mmol/l, pH 6.0). Endogenous peroxidase was inhibited by 0.3% H_2_O_2_, and non‐specific protein binding was blocked with 10% goat serum. The sections were then incubated with primary antibody against AFP and CXCR4 (1:100 dilution; Santa Cruz Biotechnology Inc., Santa Cruz, CA, USA) at 4°C overnight. Non‐immune immunoglobulin G was used as a negative control, and antigenic sites were localized using a SP9000 Polymer Detection System and a 3,3′‐diaminobenzidine kit (ZSGB‐BIO, Beijing, China).

### Cell culture

The AFP‐producing human HCC cell line Bel 7402 and the non‐AFP‐producing human HCC cell line HLE 18 were the gifts from the Department of Cell Biology, Peking University Health Science Center (Beijing, China), and were grown in DMEM (Gibco, Carlsbad, CA, USA) supplemented with 10% foetal calf serum (FCS) (Gibco), and 100 U/ml penicillin and 100 μg/ml streptomycin. All cell lines were cultured at 37°C in a humidified atmosphere with 5% CO_2_.

### RNA interference

For the RNA interference (RNAi) experiments, the anti‐AFP‐specific siRNA‐expressed vectors (AFP‐siRNA) directed at the 923–944 region of the *AFP* gene and a corresponding scrambled sequence as the negative control were used in this study as described previously 17, 19. The transfection of AFP‐siRNA vectors into Bel 7402 cells were induced by Lipofectamine 2000 (Invitrogen, Carlsbad, CA, USA).

### Generation of an AFP‐expressed construct

The AFP‐expressed construct (pcDNA3.1‐*afp*) was created as described previously 19. The transient transfections were conducted in non‐AFP‐producing HLE Cells (1 × 10^5^ cells/well in a 12‐well plate for a confluent cell layer) using Lipofectamine 2000 (Invitrogen) in Opti‐MEM reduced serum medium (Invitrogen).

### Wound healing assay

Cells motility was analysed by a wound healing assay. One day before scratching, HLE cells were transfected with pcDNA3.1‐*afp* vectors and Bel 7402 cells were transfected with AFP‐siRNA vectors; the cells were seeded into 12‐well plates to almost total confluence in 24 hrs. A scratching wound was made by scraping the middle of the cell monolayer with a sterile micropipette tip. After all detached cells were washed away with PBS, the cells were cultured with medium containing 10% FCS, images of the cells migrating into the wound area were captured at 0, 24, 48 and 72 hrs by an inverted microscope (100×) and their distances were recorded. Cell‐repaired motility was evaluated using the following formula: Cell repair ratio (%) = (distance 0 hour − distance X hours)/distance 0 hour × 100% (X: observed time points).

### Cell migration and invasion assay

Cell migration and invasion assays were carried out according to the manufacturer's protocols. To measure cell migration, transwell chambers were used to observe cultured cells inserts (Transwell chamber; 8‐mm pore size; Costar, High Wycombe, UK). The cells were placed into the wells of 12‐well cultured plates and the upper and lower chambers were separated. Cells (5 × 10^4^) were added to the upper chamber and cultured with serum‐free DMEM medium, whereas the lower chamber was filled with complete medium (contain 20% FCS). After 24, 48 and 72 hrs of incubation, the cells in the upper chamber were carefully removed with a cotton swab and those that had migrated through the membrane to the lower surface were fixed with 90% methanol and stained with 0.1% crystal violet. The number of cells that had migrated through the pores was quantified by counting five independent visual fields under the microscope (Olympus) using a 20× objective. For invasion assays, transwell chambers were covered with matrigel (BD Falcon, NJ, USA). The experimental procedure is similar to that for the migration assays. Three independent assays were performed.

### Xenograft to observe metastasis of HCC cells *in vivo*

The procedure protocols were performed according to previously research 5. Briefly, HLE cells and Bel 7402 cells were transfected with pcDNA3.1‐*afp* vectors and AFP‐siRNA vectors for 48 hrs respectively; then, the cells were implanted into the left or right leg fat pads (2 × 10^5^ cells) of 8‐week‐old male severe combined immune deficiency (SCID) mice. Then 14 days after inoculation, CdTe/Cds quantum dots of biolabelled AFP (Zhongding Corp, Shenzhen, China) were injected into the lateral tail veins of the mice and allowed to incubate for 2 hrs. Xenogen VISI Lumina II instrument (Cold Spring Biotech Corp., NY, USA) was then used to observe the locations of malignant liver cells based on methods described by Wang *et al*. 20. Animal handling procedures were approved by the Hainan Medical College Institutional Animal Care and Use Committee.

### Western blotting analysis

The AFP‐producing Bel 7402 cells were transfected with AFP‐siRNA vectors and HLE cells were transfected with pcDNA3.1‐*afp* vectors for 6 hrs and the medium was replaced with complete medium for 18, 42 or 66 hrs. Western blotting assay was performed at these time points. Briefly, total proteins were extracted using RIPA lysis buffer (Beyotime Institute of Biotechnology, Jiangsu, China). The proteins (50 μg total) were subjected to SDS‐PAGE and transferred to polyvinylidene fluoride membranes. After incubating with 5% skim milk in Tris‐buffered saline and Tween‐20 (TBST) at 37°C for 30 min, the membranes were probed for the following primary antibodies: mouse anti‐AFP (1:500), k19 (1:500) or β‐actin (1:1000); rabbit ant‐EpCAM (1:500), ‐MMP2/9 (1:400) or ‐CXCR4 (1:400) antibody (all from Santa Cruz Biotechnology Inc.) overnight at 4°C. After three washes with TBST, the membranes were incubated with horseradish peroxidase‐conjugated secondary antibodies for 1 hr at 37°C. The bands were visualized using enhanced chemiluminescence reagents (Thermo Fisher, Rockford, IL, USA) and analysed with a gel analysis system (VersDoc TM5000MP System; Bio‐Rad, Guangzhou, China). The expression of β‐actin was used as loading control.

### Statistical analysis

The results of multiple observations are presented as mean ± S.D. of at least three independent experiments. Statistical significance was determined using Student's *t*‐test and anova statistics (*F*‐test) (SPSS 11.5 software for Windows; SPSS Inc., Chicago, IL, US).

## Results

### High serum concentration of AFP positively correlated with metastasis of clinical HCC patients

**Figure 1:**
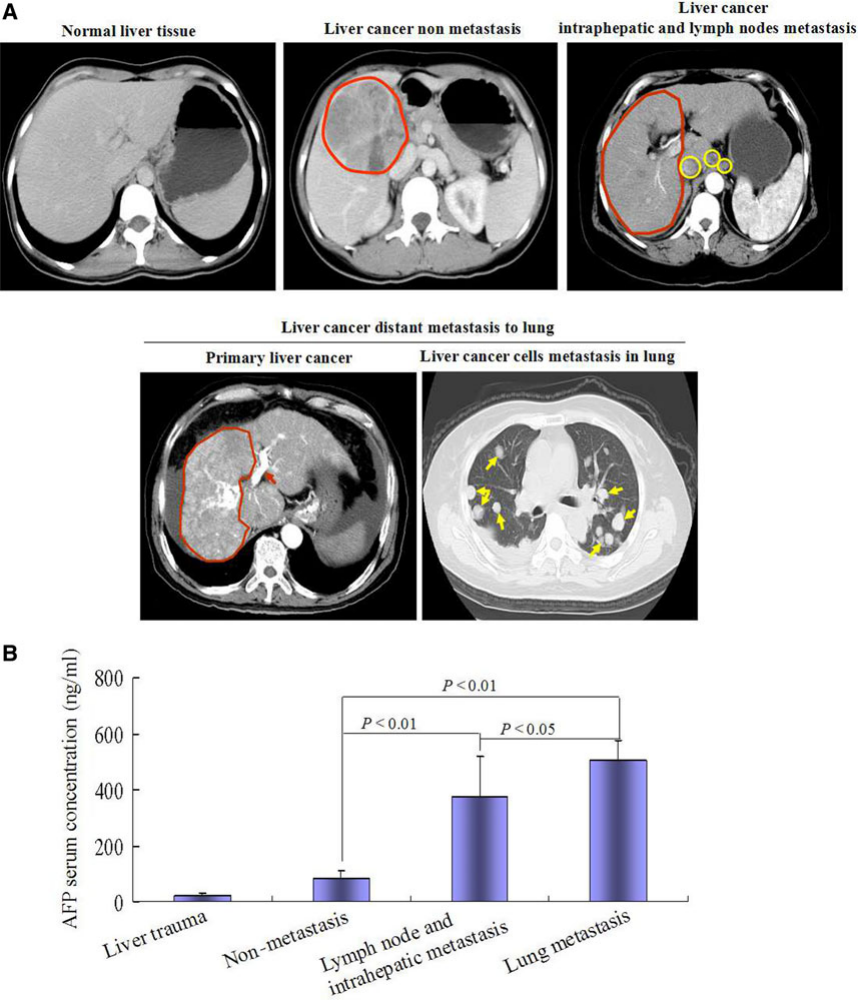
High serum concentration of AFP was positively correlated with metastasis in HCC patients. (**A**) The human normal (six cases liver trauma) and HCC patient (41 cases) livers were scanned by computerized tomography (CT); red region indicated the tumour, yellow region indicated nidus of metastasis; red arrow indicated liver cancer cells embolism in hepatic vein, yellow arrow indicated liver cancer cells metastasis to lung. (**B**) Serum concentration of AFP in the patients was detected by ELISA. The images were a representation of CT scanned photographs.

In present investigation, ELISA was used to detect serum concentration of AFP in all normal cases (liver trauma) and clinical HCC patients. Intrahepatic metastasis, nidus metastasis and lung metastasis of HCC were confirmed by CT (Fig. 1A). The results indicated that serum concentration of AFP in liver trauma patients less than 40 ng/ml, and (84.0 ± 27.0) ng/ml in non‐metastasis HCC patients, (374.0 ± 145.9) ng/ml in intrahepatic or nidus metastasis HCC patients and (506.6 ± 89.4) ng/ml in lung metastasis HCC patients. Serum AFP concentration was significantly higher in metastatic HCC patients than that in liver trauma and non‐metastasis HCC patients (*P* < 0.01) (Fig. 1B). The results showed that high serum concentration of AFP positively correlated with metastasis of HCC patients (Table S1).

### High level expression of AFP and metastasis‐related proteins in metastatic HCC patients’ liver tissue samples

**Figure 2:**
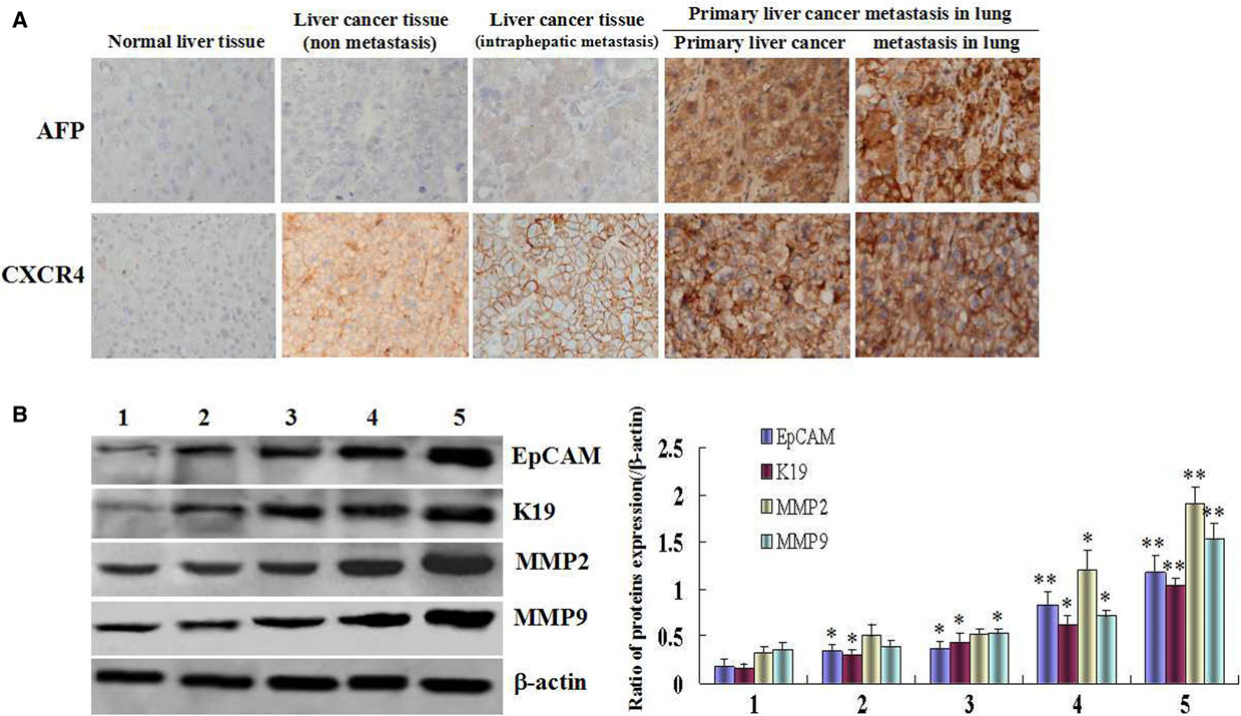
The expression profiles of AFP, CXCR4, EpCAM, K19, MMP2 and MMP9 in metastatic HCC tissue specimens. (**A**) The expression of AFP and CXCR4 in the liver tissue samples were detected by immuno‐histochemistry. (**B**) The expression of EpCAM, K19, MMP2 and MMP9 in the liver tissue samples were detected by Western blotting; left column graph indicated the relative quantity of protein expression in the tissues. (1) Normal liver tissue; (2) liver cancer tissue (non‐metastasis); (3) liver cancer tissue (intraphepatic metastasis); (4) primary liver cancer which metastasis to lung (primary liver cancer); (5) tissue of liver cancer metastasis in lung. The images were representation of three independent analysis. *P < 0.05 *versus* normal liver tissue groups, and **P < 0.01 *versus* normal liver tissue and non‐metastasis HCC groups. The images were a representation of three independent experiments.

Immunohistochemical analysis indicated that expression of AFP and CXCR4 elevated significantly in the metastatic HCC patients’ tissue samples than that in the liver trauma (normal liver tissues) and non‐metastatic HCC patients (Fig. 2A); Western Blotting assay also showed that expressions of metastasis‐related proteins, including EpCAM, K19, MMP2/9, were increased in metastatic HCC patients’ tissues samples than that in liver trauma liver tissues samples (normal liver tissues) and non‐metastasis HCC patients tissues samples (Fig. 2B). These results implicated that high expression of AFP was associated with expression of metastasis‐related proteins in HCC patients.

### AFP promoted scratch repair of HCC cells

**Figure 3:**
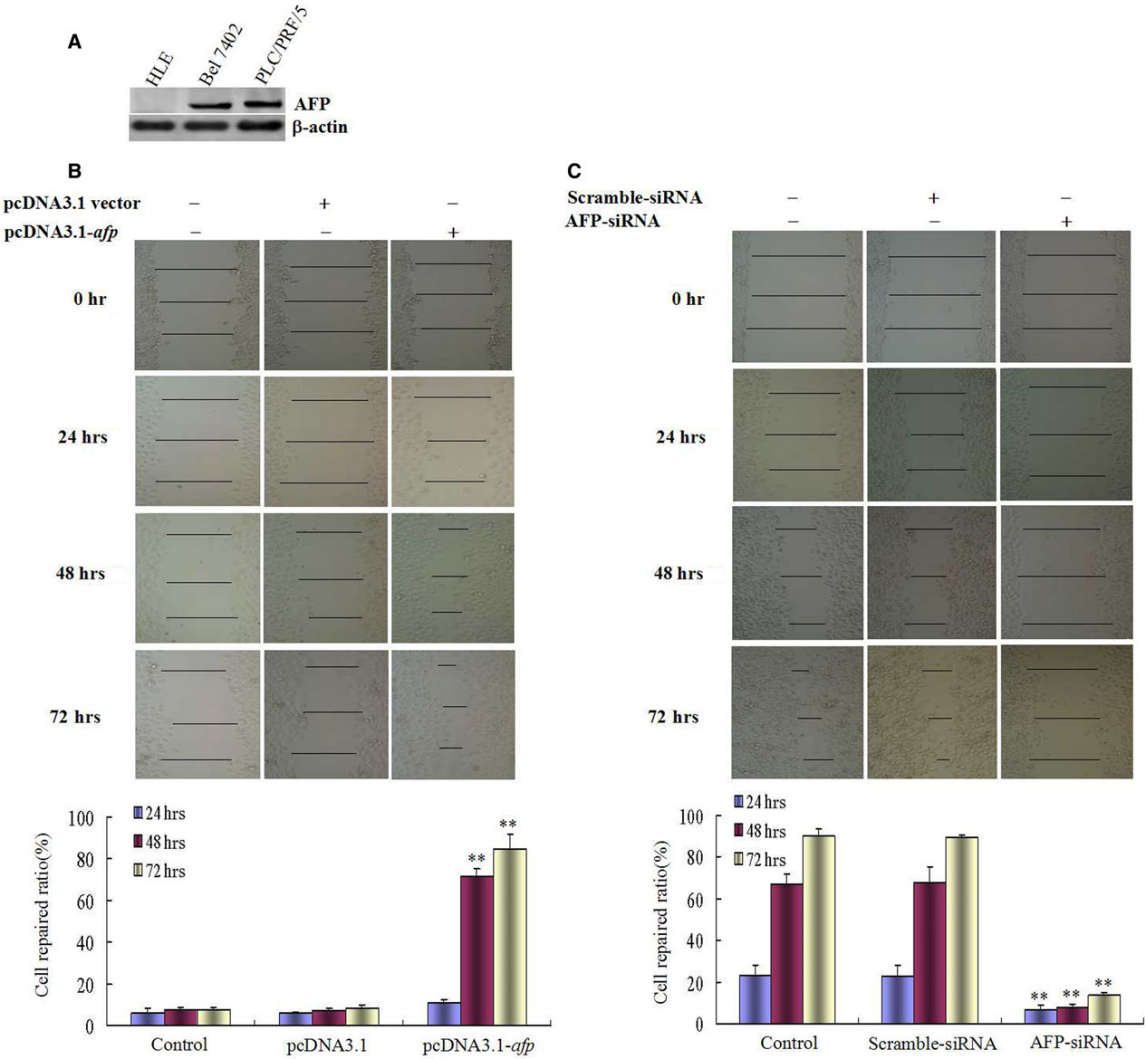
Effects of AFP on wound heal of human liver cancer cells HLE and Bel 7402 cells. (**A**) Expression of AFP in HLE and Bel 7402 cells was detected by Western blotting, PLC/PRF/5 cells were used as a positive control of AFP expressed. (**B**) HLE cells were transfected with pcDNA3.1‐*afp* vectors for 24, 48, 72 hrs, respectively; wound healing of the cells were observed by microscopy; low columnar graph showed repaired‐ratio of the cells; **P < 0.01 *versus* control and pcDNA3.1‐vectors groups. (**C**) Bel 7402 cells were transfected with AFP‐siRNA vectors for 24, 48, 72 hrs respectively; wound healing of the cells were observed by microscopy; low columnar graph showed repaired‐ratio of the cells; **P < 0.01 *versus* control and scramble‐siRNA groups. We performed three independent experiments.

In order to evaluate the effect of AFP on scratch repair of HCC cells, human hepatoma cells line, HLE (non‐AFP producing) and Bel7402 cells (AFP producing) were used as the model in this study. Wound healing assay indicated that repaired‐migration of HLE cells was significantly enhanced while HLE cells were transfected with AFP‐expressed vectors (pcDNA3.1‐*afp*) for 24, 48 and 72 hrs; the cells covered more than 80% of the scratch area at 72 hrs contrast with control cell and empty vectors (*P* < 0.05) (Fig. 3A). We also employed the scratch assay to evaluate whether AFP affected scratch repair of Bel 7402 cells. The cells were transfected with AFP‐siRNA vectors for 24, 48 and 72 hrs. A significantly decreased in scratch‐repaired capacity of the cells were observed after performed the scratch; the cells covered only 24% of the scratch area whereas control cells migrated and covered more than 80% scratch area. In contrast with control cells, the scratch‐repaired capacity of Bel 7402 cells were significantly decreased while transfected with AFP‐siRNA (*P* < 0.05) (Fig. 3B).

### AFP harbours a function to promote migration and invasion of HCC cells *in vitro*

**Figure 4:**
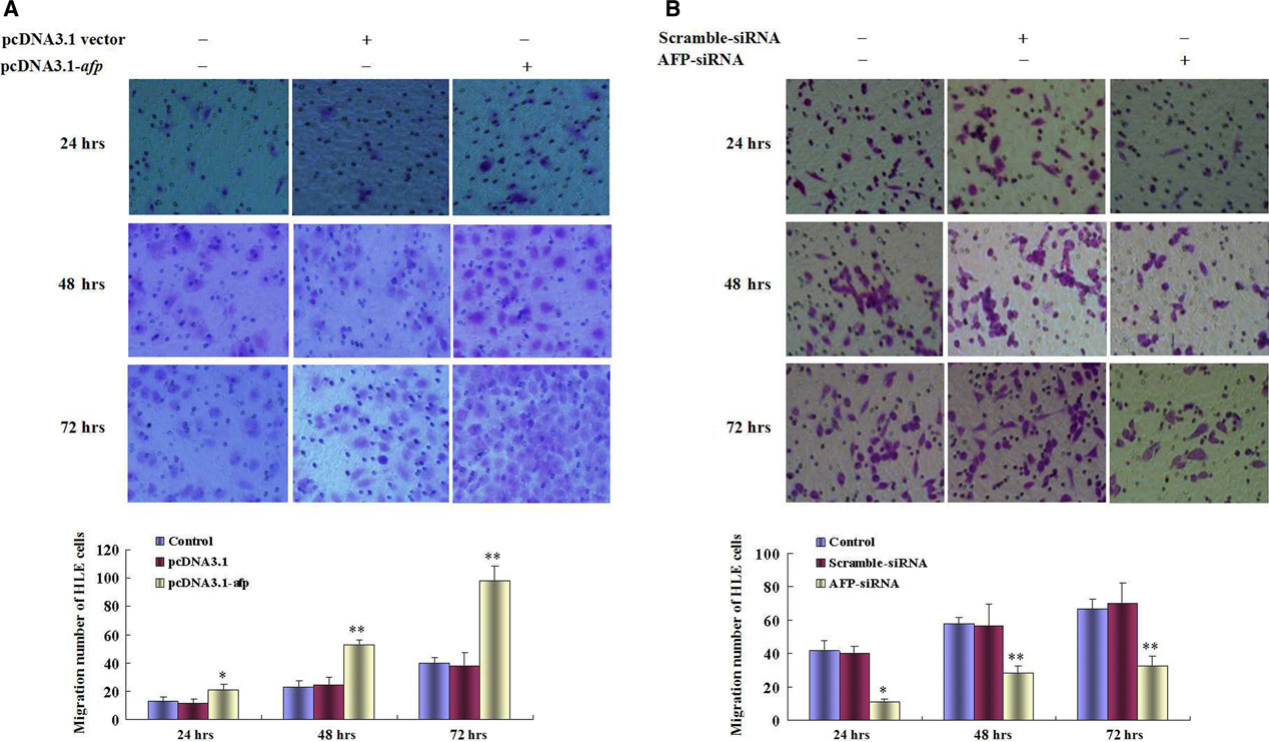
Effects of AFP on the migration of human liver cancer cells HLE and Bel 7402 cells. (**A**) HLE cells were transfected with pcDNA3.1‐*afp* vectors for 24, 48, 72 hrs respectively; migratory cells were stained with 0.1% crystal violet and observed by microscopy; low columnar graph indicated the quantity of migratory cells. *P < 0.05, **P < 0.01 *versus* control and pcDNA3.1‐vector groups. (**B**) Bel 7402 cells were transfected with AFP‐siRNA vectors for 24, 48, 72 hrs respectively; migratory cells were stained with 0.1% crystal violet and observed by microscopy; low columnar graph indicated the quantity of migratory cells; *P < 0.05, **P < 0.01 *versus* control and scramble‐siRNA groups. Three independent experiments were performed for these data.

**Figure 5:**
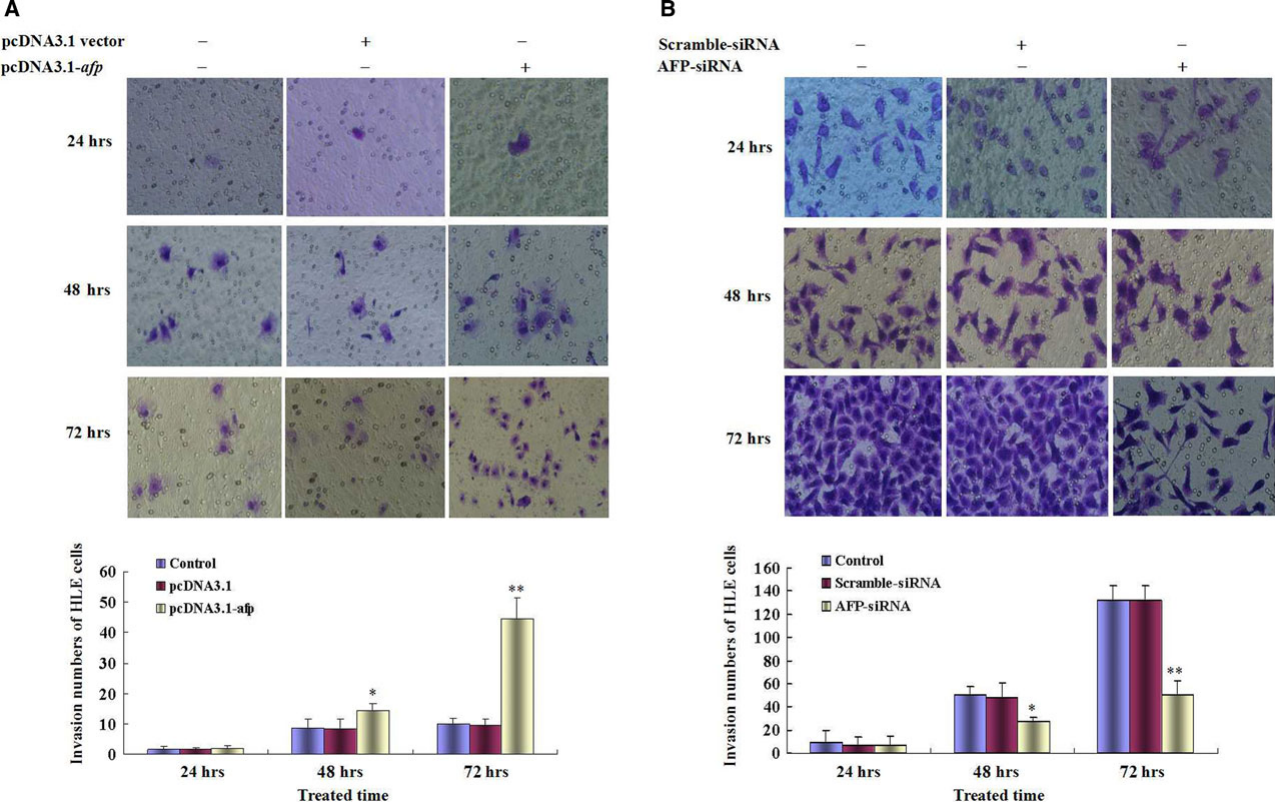
Effects of AFP on invasion of human liver cancer cells HLE and Bel 7402. (**A**) HLE cells were transfected with pcDNA3.1‐*afp* vectors for 24, 48, 72 hrs, respectively; invasive cells were stained with 0.1% crystal violet and observed by microscopy; low columnar graph indicated the quantity of invasive cells; *P < 0.05, **P < 0.01 *versus* control and pcDNA3.1‐vector groups. (**B**) Bel 7402 cells were transfected with AFP‐siRNA vectors for 24, 48, 72 hrs, respectively; invasive cells were stained with 0.1% crystal violet and observed by microscopy; low columnar graph indicated the quantity of invasive cells; *P < 0.05, **P < 0.01 *versus* control and scramble‐siRNA groups. Three independent experiments were performed for these data.

Scratch assay could not fully evaluate metastasis of HCC cells. In this study, we performed a transwell chamber assay which could prove that AFP influenced on the metastasis of HCC cells. Migration assay indicated that through‐pores capacity of HLE cells were significantly enhanced while transfected with pcDAN3.1‐*afp* vectors at 24 hrs, and persisted reinforcement at 72 hrs, in contrast to control cells and empty vectors (*P* < 0.05) (Fig. 4A). However, the through‐pores capacity of Bel 7402 cells were significantly decreased while transfected with AFP‐siRNA at 24 hrs and persisted reduction at 72 hrs contrast to control cells and scramble vectors (*P* < 0.05) (Fig. 4B). Invasion assay also showed that the capacity of HLE cells to penetrate matrigel in transwell chamber significantly strengthened while transfected with pcDNA3.1‐*afp* vectors in contrast to control cells and empty vectors (*P* < 0.05) (Fig. 5A). Bel 7402 cells have a character to penetrate matrigel in transwell chamber; however, while the cells were transfected with AFP‐siRNA vectors, the capacity of the cells to penetrate matrigel were significantly descended in contrast to control cells and scramble vectors (*P* < 0.05) (Fig. 5B). These results indicated that AFP harbours a function to promote migration and invasion of HCC cells *in vitro*.

### AFP promotes distant metastasis of HCC cells *in vivo*

**Figure 6:**
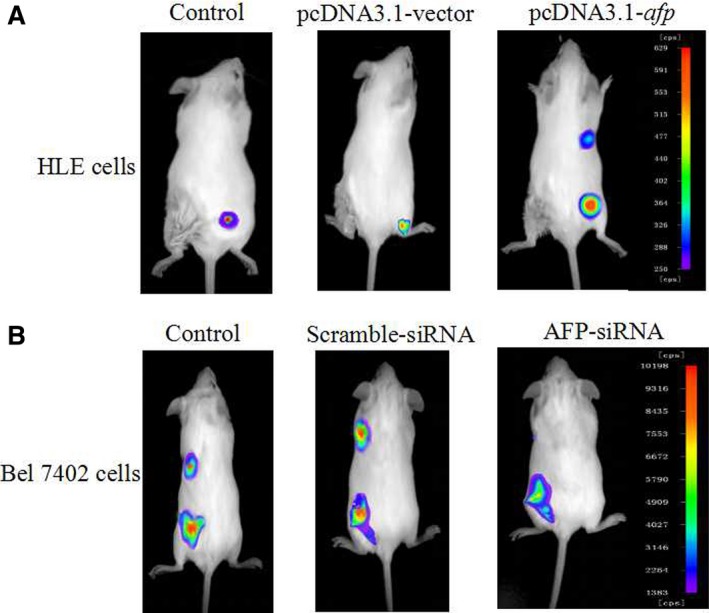
Effects of AFP on distant metastasis of HCC cells. (**A**) HLE cells were transfected with pcDNA3.1‐*afp* vectors; (**B**) Bel 7402 cells were transfected with AFP‐siRNA vectors. The cells (2 × 10^5^) were implanted into SCID mice for 14 days; CdTe/Cds quantum dots of biolabelled AFP were injected into the lateral tail veins of the mice, then Xenogen VISI Lumina II instrument was used to observe location of HCC cells. These images were a representation of six independent experiments.

To observe the effects of AFP on metastasis of HCC cells, the HLE cells were transfected with pcDNA3.1‐*afp* vectors, and the Bel 7402 cells were transfected with AFP‐siRNA vectors. The effects of these vectors on expression of AFP, AFP receptors (AFPR), pAKT (Ser473) and CXCR4 were detected by Western blotting before xenograft into SCID mouse. The results indicated the AFP overexpression and stimulated the expression of AFPR, pAKT (Ser473), CXCR4 in HLE cells while transfected with pcDNA3.1‐*afp* vectors for 48 hrs (Fig. S1A), whereas expression of AFP was significantly inhibited and expression of AFPR, pAKT (Ser 473) and CXCR4 were also repressed in Bel 7402 cells while transfected with AFP‐siRNA for 48 hrs (Fig. S1B). The distant metastasis emerged of HLE cells was observed by Xenogen VISI Lumina II instrument after the cells were xenografted into SCID mouse for 14 days (Fig. 6A); the results also showed that control Bel 7402 cells appeared distant metastasis but tiny metastasis were occurred in Bel 7402 cells which carried AFP‐siRNA vectors (Fig. 6B). These resulted demonstrated that AFP has a capability to promote distant metastasis of HCC *in vivo*.

### AFP stimulates expression of metastasis‐related proteins in HCC cells

**Figure 7:**
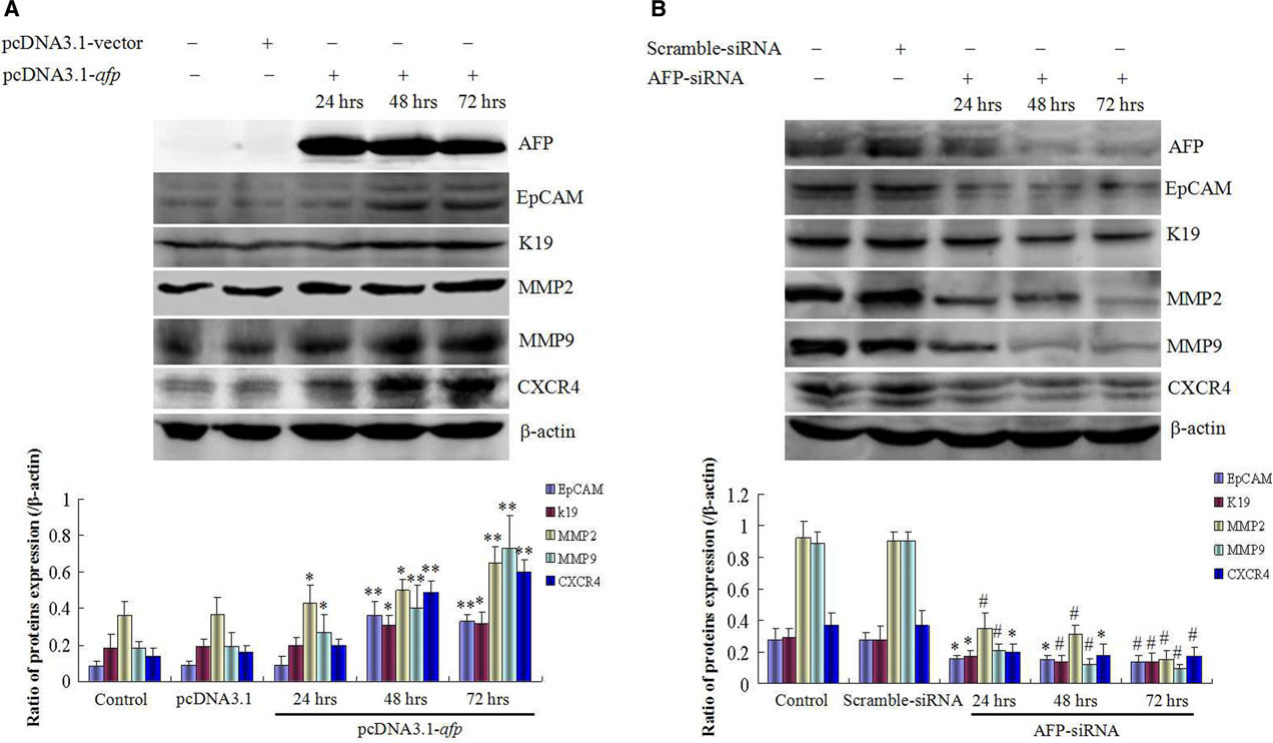
Effects of AFP on expression of metastasis‐associated proteins in human liver cancer cells HLE and Bel 7402. (**A**) HLE cells were transfected with pcDNA3.1‐*afp* vectors for 24, 48, 72 hrs, respectively; expression of AFP, EpCAM, K19, MMP2, MMP9 and CXCR4 was observed by Western blotting; low columnar graph indicated the relative quantity of protein expression; *P < 0.05, **P < 0.01 *versus* control and pcDNA 3.1‐vectors groups. (**B**) Bel 7402 cells were transfected with AFP‐siRNA vectors for 24, 48, 72 hrs, respectively; expression of AFP, EpCAM, K19, MMP2, MMP9 and CXCR4 was evidenced by Western blotting; low columnar graph indicated the relative quantity of protein expression in the cells; *P < 0.05, ^#^ *P* < 0.01 *versus* control and scramble‐siRNA groups. These images were a representation of three independent experiments.

The metastasis of cancer cells involved in the expression of metastasis‐related proteins, including EpCAM, K19, MMPs and CXCR4 9, 10, 11, 12. To explore the molecular mechanism of AFP‐promoted metastasis of HCC cells, we performed the investigation of AFP effects on the expression of these proteins. Western blotting analysed indicated that AFP overexpression in HLE cells while transfected with pcDNA3.1‐*afp* vectors for 24 hrs and persist the enhancement for 48 and 72 hrs. Expression of EpCAM, K19, MMP2/9 and CXCR4 was significantly stimulated concomitant with overexpression of AFP contrast to the control cells and empty vectors (*P* < 0.05) (Fig. 7A). Bel 7402 cells, an AFP‐producing HCC cells line, Western blotting assay showed that EpCAM, K19, MMP2/9 and CXCR4 also expressed in this cells, but expression of AFP was repressed while the cells were transfected with AFP‐siRNA vectors for 24 hrs and persisted descending for 48 and 72 hrs, expression of EpCAM, K19, MMP2/9 and CXCR4 proteins were significantly decreased concomitant with expression repressed of AFP contrast to control cells and scramble vectors (*P* < 0.05) (Fig. 7B). These results confirmed that AFP stimulated the expression of metastasis‐related genes in HCC cells.

## Discussion

Alpha fetoprotein, a novel member of regulation the signal transduction involve in cancer cells malignant behaviours was discovered recently 17, 21, but whether AFP plays a role in promoting metastasis of HCC cells remains poorly understood. In this study, we elucidated that high serum concentration of AFP was positively associated with metastasis of HCC cells in clinical patients, and found that AFP harbours a function to promote metastasis of liver cancer cells *in vitro* and *in vivo*. Our clinical data showed that AFP (>40 ng/ml) expression in all of HCC metastatic specimens from 100% (25/25) HCC patients, and 93.7% (15/16) AFP expression in non‐metastatic HCC patients. The serum concentration of AFP in metastatic HCC patients significantly elevated than that in non‐metastatic HCC patients (*P* < 0.01). Significantly, high levels of metastasis‐related proteins associated with AFP expression in metastasis of clinical HCC patients. These results indicated that elevated levels of AFP closely associated with the metastasis of HCC cells.

*AFP* gene expressed was reactivated in the early stage of hepatocarcinogenesis. Documents evidenced that metastasis of cancer cells maybe occurred concomitant with carcinogenesis 22, implicated that expression of AFP in early stage of hepatocarcinogenesis involved in malignant behaviours of liver cancer cells. The data of investigations revealed that AFP was not only promoted proliferation of HCC cells 23, 24, 25, 26 but also played a critical role in hepatocarcinogenesis 27. Previously, we had found that extracellular AFP was able to stimulate the growth of HCC cells or other cancer cells, which was mediated by AFPR 8, 28; cytoplasmic AFP blocked retinoic acid receptor β interaction with the promoter of fibroblast growth factor‐inducible 14 (Fn14) gene to promote the expression of Fn14, and led to HCC cells antagonist all *trans* retinoic acid to induce apoptosis 29; these results exhibited that the intracellular or extracellular AFP contributed to malignant behaviours of HCC cells. Although other studies had found that the expression of AFP was closely associated with hepatocarcinogenesis, the role of AFP in the metastasis of HCC cells was still unclear. In this investigation, human liver cancer cells HLE and Bel 7402 cells were selected for transfection with AFP overexpression vectors (pcDNA3.1‐*afp*) and silenced vectors (AFP‐siRNA), respectively; both scratch assay and transwell chamber assay indicated that overexpression of AFP in HLE cells was able to significantly repair the scratch area and penetrate the transwell chamber pores in contrast with control cells; whereas the capacity of repairing, migration and invasion of Bel 7402 cells was significantly declined while transfected with AFP‐siRNA vectors. To estimate effects of AFP on the metastasis of HCC cells, we performed the xenograft experiment, in which the SCID mice were implanted with HLE cells that carried pcDNA3.1‐*afp* vectors or Bel 7402 cells that carried AFP‐siRNA vectors. The results indicated that HLE cells (carried pcDNA3.1‐*afp* vectors) possessed a metastatic capability contrast with control cells; however, in Bel 7402 cells which carried AFP‐siRNA vectors, the metastatic capacity was significantly descended in comparison to the control cells. These results revealed that AFP played critical role in promoting metastasis of HCC cells *in vitro* and *in vivo*.

Expression of metastasis‐related proteins contributed to metastasis of cancer cells. EpCAM is not only a tumour biomarker of HCC 30, 31 but also an important molecule for promoting metastasis of HCC cells 32; K19, MMP2/9 and CXCR4 also played a pivotal role in promoting metastasis of HCC cells 9, 11, 15, 33, 34. The expressions of these proteins were regulated by PI3K/AKT signal pathway 5, 17. In our previous studies, we had found that AFP inhibited activity of PTEN to activate PI3K/AKT signal pathway to stimulate expression of Ras, Src and CXCR4 5, 17, 35, 36, and AFP promoted malignant behaviours of HCC cells also through regulating post‐transcription of PTEN 37. In this study, we found that AFP promoted expression of metastasis‐related proteins, implicated that AFP stimulated expression of these proteins possibly *via* activating PI3K/AKT signal. We conferred that the molecular mechanism of AFP promoted metastasis of HCC cells that involved in activating transduction of PI3K/AKT signal pathway.

In conclusion, this report is the first time to demonstrate that AFP promoted HCC cells metastasis directly. AFP overexpression in HCC cells was related to metastatic characteristics in human HCC patients. Overexpression of AFP plays a critical role in promoting invasion and distant metastasis of HCC cells through up‐regulating expression of metastasis‐related proteins. The molecular mechanism of AFP promoted metastasis of HCC cells *via* activating PI3K/AKT signal pathway. Thus, AFP could be applied as a novel therapeutic target for treating HCC patients.

## Conflicts of interest

The authors declare that there is no conflict of interest in this work.

## Supporting information


**Figure S1** Effects of AFP in expression of AFP receptor (AFPR), pAKT (Ser 473) and CXCR4 in human heaptoma cells.
**Table S1** Data of clinical patients.
