# Supplementary material for: Alpha fetoprotein plays a critical role in promoting metastasis of hepatocellular carcinoma cells
Source: J Cell Mol Med. 2016 Jan 12;20(3):549–58. doi: 10.1111/jcmm.12745 (PMC4759472; doi:10.1111/jcmm.12745)
Supplement: Supplementary file 1 — Figure S1 Effects of AFP in expression of AFP receptor (AFPR), pAKT (Ser 473) and CXCR4 in human heaptoma cells. Table S1 Data of clinical patients. [file JCMM-20-549-s001.doc]

**Supplement materials**

Table 1. Data of clinical patients

| No. | sex^a^ | Age(years) | Diagnosis^b^ | AFP concentration(ng/ml)^c^ | metastasis^d^ |
| --- | --- | --- | --- | --- | --- |
| 1 | M | 36 | liver trauma | 9.8 | N |
| 2 | F | 54 | liver trauma | 12.7 | N |
| 3 | M | 48 | liver trauma | 14.6 | N |
| 4 | M | 52 | liver trauma | 6.8 | N |
| 5 | F | 33 | liver trauma | 21.7 | N |
| 6 | M | 55 | liver trauma | 23.5 | N |
| 7 | M | 59 | HCC | 83.4 | N |
| 8 | M | 47 | HCC | 35.5 | N |
| 9 | F | 33 | HCC | 102.8 | N |
| 10 | M | 69 | HCC | 92.6 | N |
| 11 | M | 47 | HCC | 107.9 | N |
| 12 | F | 51 | HCC | 75.2 | N |
| 13 | M | 54 | HCC | 125.6 | N |
| 14 | M | 36 | HCC | 77.8 | N |
| 15 | M | 42 | HCC | 65.4 | N |
| 16 | F | 47 | HCC | 83.8 | N |
| 17 | M | 63 | HCC | 93.7 | N |
| 18 | F | 72 | HCC | 74.7 | N |
| 19 | F | 56 | HCC | 89.3 | N |
| 20 | M | 41 | HCC | 55.8 | N |
| 21 | M | 46 | HCC | 141.5 | N |
| 22 | M | 38 | HCC | 49.8 | N |
| 23 | F | 53 | HCC | 194.7 | intraphepatic and lymph nodes |
| 24 | F | 71 | HCC | 328.1 | intraphepatic and lymph nodes |
| 25 | M | 65 | HCC | 452.2 | intraphepatic and lymph nodes |
| 26 | M | 53 | HCC | 526.8 | intraphepatic and lymph nodes |
| 27 | F | 47 | HCC | 358.4 | intraphepatic and lymph nodes |
| 28 | M | 56 | HCC | 289.5 | intraphepatic and lymph nodes |
| 29 | F | 37 | HCC | 165.3 | intraphepatic and lymph nodes |
| 30 | M | 31 | HCC | 499.7 | intraphepatic and lymph nodes |
| 31 | M | 48 | HCC | 363.6 | intraphepatic and lymph nodes |
| 32 | M | 63 | HCC | 628.4 | intraphepatic and lymph nodes |
| 33 | M | 43 | HCC | 322.5 | intraphepatic and lymph nodes |
| 34 | M | 59 | HCC | 443.2 | intraphepatic and lymph nodes |
| 35 | F | 51 | HCC | 265.9 | intraphepatic and lymph nodes |
| 36 | M | 62 | HCC | 287.7 | intraphepatic and lymph nodes |
| 37 | M | 77 | HCC | 589.6 | metastasis in lung |
| 38 | M | 65 | HCC | 448.8 | metastasis in lung |
| 39 | F | 48 | HCC | 392.5 | metastasis in lung |
| 40 | M | 39 | HCC | 405.6 | metastasis in lung |
| 41 | M | 41 | HCC | 535.8 | metastasis in lung |
| 42 | M | 46 | HCC | 667.4 | metastasis in lung |
| 43 | F | 53 | HCC | 431.7 | metastasis in lung |
| 44 | M | 58 | HCC | 547.4 | metastasis in lung |
| 45 | M | 47 | HCC | 483.3 | metastasis in lung |
| 46 | F | 50 | HCC | 569.5 | metastasis in lung |
| 47 | M | 49 | HCC | 506.2 | metastasis in lung |

Note: a, M: man, F: female; b, diagnosis by and pathological tissue section and CT scanned photography; c, serum AFP concentration were detected by enzyme-linked immunosorbent assay(ELISA); d, N: non metastasis.


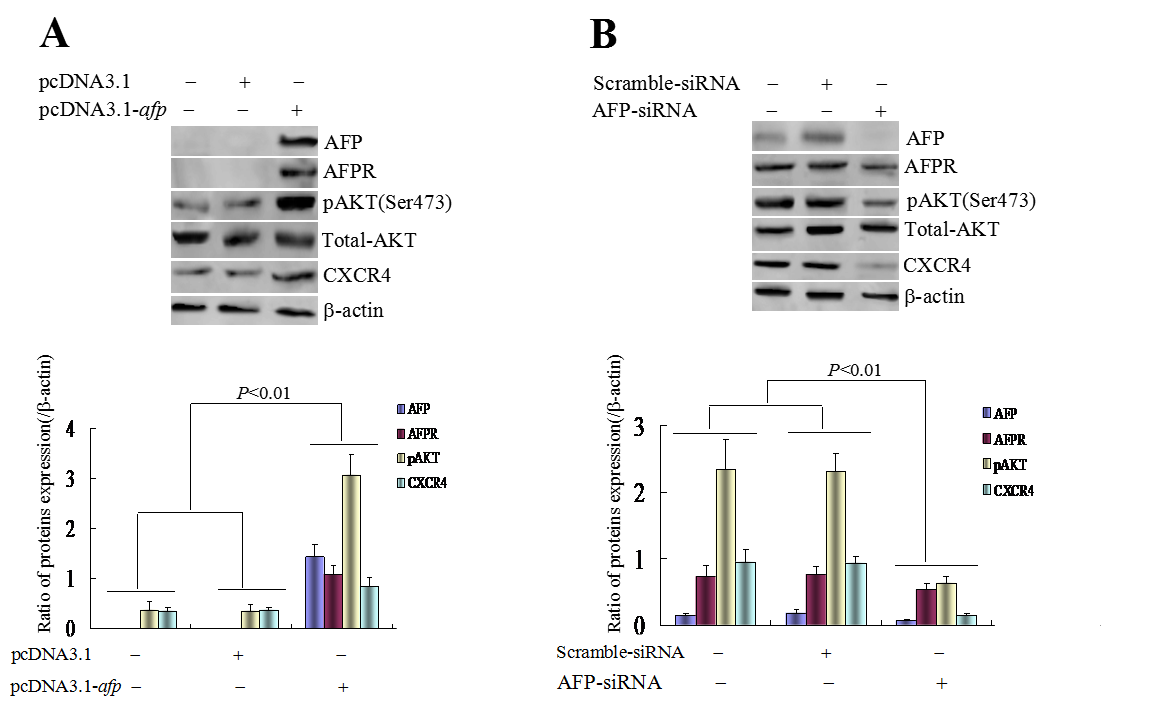


**Supplement-Figure 1**. Effects of AFP in expression of AFP receptor(AFPR ), pAKT(Ser 473) and CXCR4 in human heaptoma cells. A, HLE cells were transfection with pcDNA3.1-*afp* vectors for 48 hours, expression of AFPR, pAKT(Ser 473) and CXCR4 in the cells were detected by Western blotting. B, Bel 7402 cells were transfection with AFP-siRNA vectors for 48 hours, expression of AFPR, pAKT(Ser 473) and CXCR4 in the cells were detected by Western blotting. The images were representation of three independent experiments.
